# Assessment of two treatment protocols using prebent titanium mesh and customized PEEK mesh for predominantly horizontal maxillary ridge augmentation with volumetric evaluation: a randomized clinical trial

**DOI:** 10.1186/s12903-026-08720-w

**Published:** 2026-06-16

**Authors:** Basma Abd Alkader Alshikh, Mohamed Hassan Eid, Amr Amin, Mohamed Elsholkamy

**Affiliations:** 1 Oral and Maxillofacial Surgery Department, Faculty of Dentistry, Suez University, Suez City, Egypt; 2 Oral and Maxillofacial Surgery Department, Faculty of Dentistry, The British University in Egypt, El Sherouk City, Egypt

**Keywords:** Alveolar Ridge Augmentation, Guided Bone Regeneration, Titanium Mesh, Bone Regeneration

## Abstract

**Background:**

Reconstruction of the deficient maxillary ridge in three dimensions is crucial for the successful placement of implants. Prebent titanium meshes are an established modality for GBR, while patient-specific PEEK meshes have recently emerged with some possible advantages over the former. This study compared the effectiveness of two treatment protocols involving prebent titanium mesh and customized PEEK mesh, each combined with different graft compositions for bone augmentation.

**Materials and methods:**

14 patients with 28 augmented sites in the maxillary ridges of primarily horizontal bone deficiencies, often associated with minor vertical components, were randomly assigned to two groups. The control group (*n* = 7) was augmented with a prebent titanium mesh, while the study group (*n* = 7) was augmented with a customized milled PEEK mesh. Both were grafted with a mixture of autogenous bone and xenograft. The primary outcome was horizontal bone gain. Secondary outcomes included vertical bone gain, gained bone volume, and graft resorption. These parameters were assessed by CBCT preoperatively, immediately postoperatively, and at 6 months before implant placement.

**Results:**

In this comparative three-dimensional volumetric CBCT analysis of predominantly horizontal ridge augmentation, no statistically significant differences were observed between the two groups regarding horizontal or vertical bone gain (Titanium: 3.02 ± 0.68 mm vs. PEEK: 2.42 ± 0.38 mm; *p* = 0.065, and Titanium: 1.12 ± 0.10 mm vs. PEEK: 1.09 ± 0.25 mm; *p* = 0.738, respectively). Regarding secondary outcomes, the PEEK group, which received a graft composed of a higher percentage of autogenous bone (70:30 autograft: xenograft), showed a significantly higher amount of gained bone volume when compared to the titanium mesh group (who received a 60:40 graft mixture) (499.47 ± 80.46 mm³ vs. 370.82 ± 51.69 mm³; *p* = 0.004), as well as a higher graft loss volume (174.83 ± 40.78 mm³ vs. 127.73 ± 35.55 mm³; *p* = 0.040). Clinical complications, including mesh exposure, were minimal and were successfully managed in both groups.

**Conclusion:**

Both prebent titanium mesh and custom-made PEEK mesh materials were associated with successful three-dimensional maxillary ridge augmentation within the limitations of this study. Although differences in volumetric outcomes were noted, it should be emphasized that such differences may have been influenced by differences in graft composition. Therefore, the current study represents a comparison of two treatment protocols incorporating different mesh materials and graft compositions.

**Trial registration:**

The study was prospectively registered at Clinical Trials.gov (NCT07040124) on June 25, 2025, before patient enrollment.

## Introduction

Successful implant placement requires an adequate volume of alveolar bone for functional and aesthetic success. However, alveolar ridge deficiencies are a common problem that often manifests in the form of horizontal deficiencies [1–3].

Guided bone regeneration (GBR) is now a well-established procedure for ridge augmentation, especially with the use of non-resorbable materials, which provide potentially favorable space-maintaining properties [2]. The effectiveness of guided bone regeneration in both horizontal and vertical ridge augmentation has been proven in systematic reviews, although this depends on the materials and techniques used [2–4].

Titanium meshes have been popular in GBR procedures because of their rigidity, biocompatibility, and ability to provide space for bone regeneration. Nevertheless, titanium meshes have some limitations, including the need to make intraoperative adaptations and an increased risk of soft tissue complications. On the other hand, poly-ether-ether-ketone (PEEK) has been proposed as an alternative biomaterial in GBR procedures because of its favorable mechanical, radiolucent, and low tissue-irritation properties. The application of CAD/CAM technology can provide customized PEEK meshes, which may improve surgical accuracy and clinical handling. However, differences in physical properties, including porosity and permeability, can affect vascularization and bone regeneration [5].

Recently, several studies investigated the application of customized PEEK meshes in ridge augmentation procedures and compared them with titanium-based techniques. For instance, Mounir et al. (2019) [5] carried out a randomized clinical trial to compare prebent titanium and customized PEEK meshes, while Zhu et al. (2024) [6] carried out a quantitative assessment of PEEK-based meshes. Gouda et al. (2023) [7] investigated the histomorphometric results of similar techniques. The application of PEEK as a barrier membrane in GBR is gaining popularity.

However, the current level of evidence available on this topic is limited, especially in terms of volumetric evaluation of bone addition and graft resorption in a randomized clinical trial. Most of the published studies have mainly focused on linear evaluation or have not used volumetric evaluation in combination with clinical evaluation.

Several recent studies have been published on the use of customized devices for guided bone regeneration. Clinical trials and systematic evidence indicate that titanium-reinforced barriers and customized meshes can generate predictable amounts of bone gain both horizontally and vertically due to their role in space-maintenance and stabilization of the grafts [8, 9]. Moreover, histology and histomorphometry prove that besides barrier characteristics, graft composition and dynamics of vascularization are factors that affect the process of bone regeneration [10, 11].

The introduction of CAD/CAM technology to customize titanium meshes has increased accuracy and stabilization of the procedure; yet, despite favorable clinical results, there is still a lack of volumetric evidence from randomized trials comparing the use of various customized materials [12, 13].

Therefore, the current randomized clinical trial aims to further evaluate this topic by comparing two treatment protocols for ridge augmentation, involving prebent titanium mesh and customized PEEK mesh in combination with different graft compositions, using both linear and volumetric CBCT evaluation.

### Hypothesis of the study

The null hypothesis for this study is that there is no significant difference in horizontal and vertical bone gain, gained bone volume, and graft stability between alveolar ridges augmented using prebent titanium mesh and those augmented using customized PEEK mesh.

## Subjects and methods

### Sample size calculation

Sample size was calculated using the primary outcome variable, which was horizontal bone gain (mm), and data derived from a previous randomized clinical trial (Mounir et al., 2019) [5]. To calculate the sample size, a procedure was conducted by applying an independent-samples t-test using the software package G*Power version 3.1.9.7. There is no specific procedure for calculating the sample size for a mixed-effects model. It was conducted based on the primary outcome of interest, which was horizontal bone gain. In this case, the effect size (Cohen’s d) was 0.75, α was set at 0.05, and the power was set at 80%, or 1 − β = 0.80.

The primary outcome measured in this study was horizontal bone gain, which was used to determine the sample size. The secondary outcomes were vertical bone gain, gained bone volume, and graft resorption.

### Study setting

The present study included 14 patients with 28 sites of augmented maxillary ridges, primarily presenting with horizontal deficiencies, sometimes accompanied by minor vertical components, which hinder implant placement. All patients were recruited and treated at the Faculty of Dentistry, Suez Canal University, where all clinical procedures were performed.

### Ethical approval

All selected patients were informed about the details of the study and signed an informed consent. Ethical approval was obtained from the Research Ethics Committee of Suez University (Approval No. 393/2021). Although ethical approval was obtained in October 2020, patient recruitment and clinical procedures were initiated only after prospective trial registration on June 25, 2025. Patient recruitment was conducted between July 1, 2025, and January 1, 2026, with a total enrollment of 14 patients. No patient enrollment, allocation, or study-related procedures were performed before trial registration. All procedures performed in this study involving human participants were conducted in accordance with the 1964 Declaration of Helsinki and its later amendments or comparable ethical standards.

**Fig. 1:**
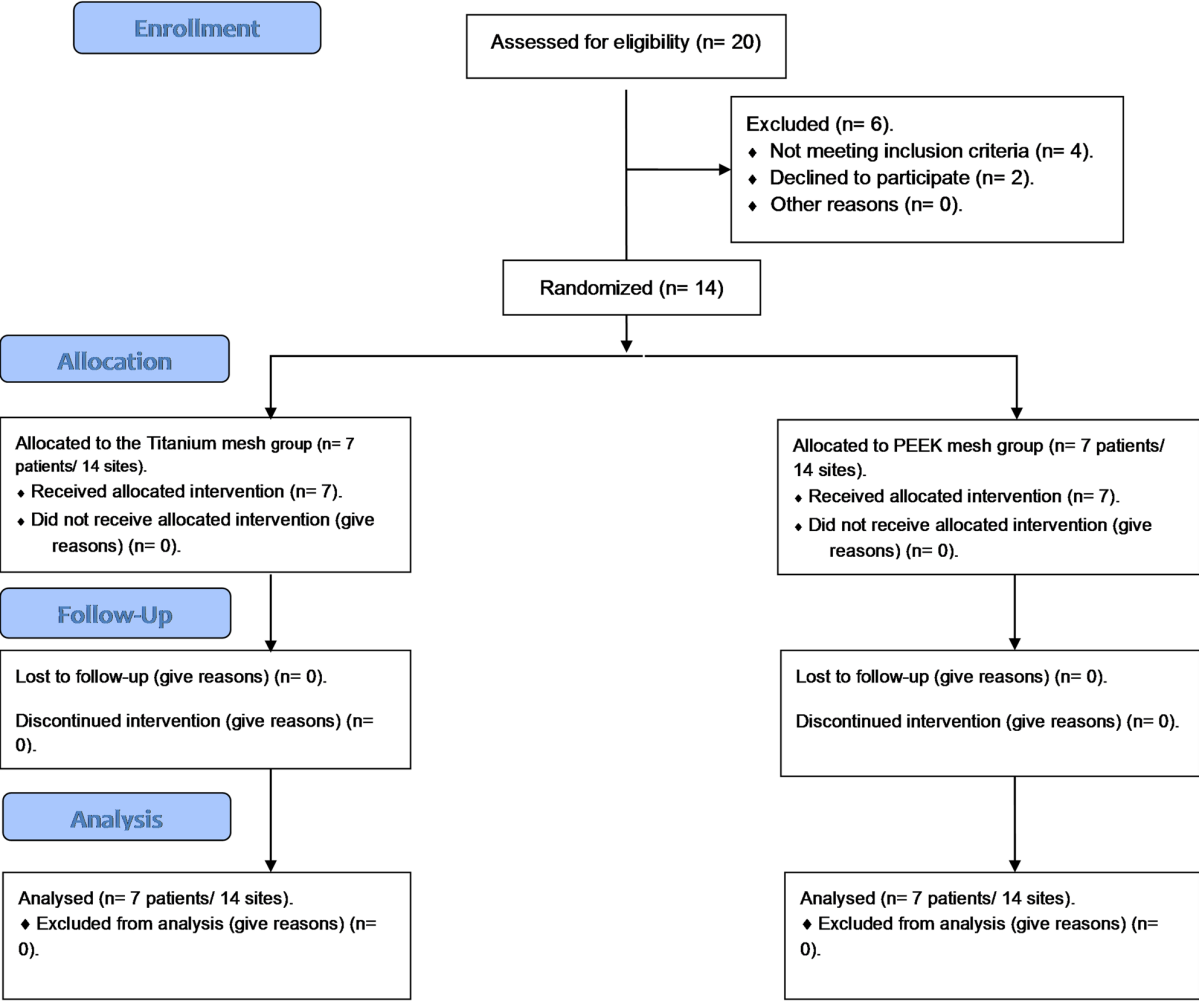
CONSORT flow diagram of participant enrollment

The Consolidated Standards of Reporting Trials (CONSORT) 2010 guidelines were followed in the conduct and reporting of this randomized clinical trial [14]. An additional file containing the completed CONSORT checklist has been provided as a supplementary file, and a CONSORT flow diagram illustrating participant enrollment, allocation, follow-up, and analysis is presented in Fig. 1.

### Randomization and patient grouping

Fourteen patients were divided randomly into two equal groups using a research randomizer software (https://www.randomizer.org/). The random allocation sequence was generated by an independent staff member who was not involved in patient recruitment, clinical procedures, or outcome assessment.

The unit of randomization was the patient (*n* = 14), and the measurements of outcome were obtained at the site level (28 augmented sites). Some patients had two sites augmented, and both sites were included in the analysis. A total of 28 sites were equally distributed between the two groups (14 sites per group). Therefore, the statistical analysis accounted for the clustering effect within patients.

Participants were included by the principal investigator based on the inclusion and exclusion criteria.

Allocation concealment was obtained through the use of sequentially numbered, opaque, sealed envelopes (SNOSE) created by the independent staff member. Envelopes included the allocated intervention and were only opened after patient recruitment, just before the surgery began.

The principal investigator assigned participants to the intervention arms based on the concealed allocation sequence.

Because more than one site was embedded within some of the patients, independence among observations on the site level could not be assumed. Thus, the nested structure of sites within patients was taken into account through mixed-models analysis.

All patients and sites that were randomized were analyzed without any losses to follow-up.

While all the included defects fulfilled the inclusion criteria according to their horizontal size, their characteristics were not stratified or otherwise compared across groups, which could be seen as a source of bias.

### Blinding

Blinding of the surgeon and the patients was not feasible owing to the nature of the procedures and the distinct physical properties of the meshes. Outcome assessment was performed by a single examiner (B.A.A). Although the CBCT scans were anonymized prior to evaluation, and group allocation was concealed during data analysis, it was not possible to blind the assessor completely due to the inherent radiographic differences between titanium and PEEK meshes.

To minimize measurement bias, all the measurements were performed twice at a two-week interval to assess the intra-examiner reliability. The reliability of the measurements was confirmed by the intraclass correlation coefficient (ICC) results, which showed good agreement (> 0.90). Statistical analysis was conducted using coded groups.

- Group 1: 7 patients with 14 augmented sites in maxillary ridges were augmented using prebent titanium mesh (served as a control group)
- Group 2: 7 patients with 14 augmented sites in maxillary ridges were augmented using patient-customized milled PEEK mesh. (served as a study group)

All patients were evaluated by CBCT preoperatively and immediately after the bone augmentation procedure, and 6 months postoperatively, before the mesh removal. All augmented ridges were evaluated for horizontal and vertical bone gain and for the ability to place implants in the augmented segments.

### Patient selection

#### Inclusion criteria

1. Adult patient of both sexes presenting with a partially edentulous maxillary ridge with a horizontal bone defect.
2. The horizontal ridge dimension measured 2 mm below the alveolar crest should range from 2 to 5 mm.
3. No local pathosis that may interfere with bone healing.
4. Good oral hygiene.
5. Age between 20 and 65 years old.

#### Exclusion criteria

1. Patient taking any medication that may interfere with normal bone physiology or impair bone healing.
2. All patients suffering from any systemic disease that may affect bone healing.
3. Heavy smokers (more than 10 cigarettes per day).
4. Patients with parafunctional habits such as bruxism and clenching.
5. Poor interest and cooperation from the patient.
6. Patients who have undergone any horizontal augmentation procedure at the site of interest.

### CBCT segmentation and volumetric analysis

All the DICOM images obtained from the CBCT scans were exported and analyzed using specific 3D image analysis software (OnDemand 3D, Cybermed, Korea). Standardization of orientation for all images was conducted prior to evaluation, in which the maxillary plane is parallel to the horizontal reference plane.

Horizontal gain of the bone was measured by standardized reference points perpendicular to the alveolar ridge, and vertical gain of the bone was compared against a fixed anatomical reference plane.

Segmentation of the augmented area was performed using a semi-automatic thresholding method. A uniform range of threshold values for all images was applied to isolate the mineralized tissue, followed by manual editing to remove artifacts and adjacent anatomical structures.

For volumetric measurements, a region of interest (ROI) was defined for the grafted area in the immediate postoperative scan, and the same ROI was duplicated for the 6-month scan. The superimposition between time points was done using voxel-based registration, focusing on stable anatomic structures such as adjacent teeth and basal bone.

The bone gain is defined as the difference in volume between the preoperative and immediate postoperative measurements, while graft resorption is defined as the difference in volume between the immediate postoperative and 6-month measurements.

### Outcome measures and CBCT analysis protocol

The primary outcome measure was the horizontal gain of bone, while the secondary outcomes involved vertical bone gain, gained bone volume, and graft resorption.

Horizontal gain of bone was determined by the linear measurement of the change in the width of the alveolar ridge in millimeters using the CBCT scan results. The width of the bone was determined perpendicularly from the center point of the ridge at the standardized reference points. Three measurements were obtained at equidistant points within the defects before averaging.

Vertical gain of bone was defined as the linear measurement of the difference in the ridge height (in millimeters) compared to the anatomical reference plane on the CBCT scan images.

Gained bone volume was measured as the volumetric difference (mm³) between the segmented grafted site on the preoperative and immediate postoperative CBCT images. Bone graft resorption was calculated as the volumetric difference (mm³) between the immediate postoperative and six months scans.

To analyze bone volume changes, all CBCT data sets were exported to 3-dimensional reconstruction software (OnDemand 3D, Cybermed, Korea). The image alignment was standardized by orienting the maxillary plane parallel to the horizontal plane.

The segmented volume of the grafted region was achieved by applying a semi-automatic segmentation algorithm based on the threshold technique, in which a consistent threshold range was used for all images to segment the mineralized tissues.

The ROI was created in the immediate postoperative image and extended to cover the full grafted site. The same ROI was then copied over to the 6-month image after registration.

The scans were aligned through voxel registration using stable anatomical landmarks such as neighboring teeth and basal bone.

In order to reduce the effect of observer bias, all image analyses were carried out by a trained single operator. Two identical measurements were taken two weeks apart, with the mean value used for further analyses. Intra- examiner reliability was assessed using the intraclass correlation coefficient (ICC) for all measurements, such as horizontal bone gain, vertical bone gain, and volumetric analysis. The ICC values demonstrated excellent agreement for all parameters (ICC > 0.90).

#### Preoperative assessment

- A thorough preoperative assessment for all enrolled candidates is carried out, including history taking, clinical, and radiographic examination.
- Medical history was taken from patients preoperatively to exclude medically compromised patients or patients with bad habits affecting osseointegration.
- Oral hygiene of the patients was assessed and referred to the Perio department to undergo scaling and polishing for the patients preoperatively.
- A preoperative digital panoramic radiograph (OPG) with 1:1 magnification has been taken for each patient as a primary survey to identify the deficient areas and exclude the presence of any lesion in the area of interest.
- A CBCT scan is ordered for the enrolled candidates to assess the extent of the defect and evaluate the ramus donor site.

#### Clinical examination

Interocclusal arch space was determined preoperatively.

Bone width was determined clinically.

#### Pre-operative radiographic evaluation

- Radiographic assessment pre-operatively by CBCT to detect the patient's vertically and horizontally deficient maxillary ridge that impairs implant placement and examine the adjacent teeth for any periapical infection. All CBCT measurements were performed by a single blinded assessor (B.A.).
- Preoperative cone beam computed tomographic radiographs using the Scanora3D imaging system, using a CMOS flat panel detector with isotropic voxel size 133 μm, the x-ray tube, which is used to scan the patients, possesses a current intensity of 10 mA, 90 kVp, and a focal spot size of 0.5mm.
- The scanning time is 14seconds of pulsed exposure, resulting in an effective exposure time of 3.2 seconds to scan the FOV (field of view) of 14 cm height ×16.5 cm width. FOV adjustment is guided by three laser light beams to centralize the area of interest within the scanning field. The primary reconstruction time for a DICOM data set is 2 minutes. Then, the raw DICOM data set images will be imported to the On-Demand software for secondary reconstruction and image analysis.
- Each patient was evaluated for bone quantity, quality, as well as the relation to the mental foramen, and the detection of the remaining roots or any suspected pathological lesions. Cone beam computed tomographic evaluation will be performed to allow for a more comprehensive overall view and better interpretation of the anatomic structures. As well as the patients who have neighboring remaining roots or carious lesions are planned for treatment.

#### Preoperative medication

All patients received strict oral hygiene instruction to maintain periodontal health in the form of oral rinses with 0.12% chlorohexidine gluconate * 3 times per day. 

Each patient was instructed to administer a prophylactic antibiotic in the form of Amoxicillin 875mg. & Clavulanic acid 125mg twice daily, one day before surgery, and continued for 5 days after surgery.

#### Surgical procedure

All the surgical procedures were performed by the same surgeon using a standardized technique under aseptic conditions. All patients were operated under local anesthesia, which was injected at the defect site (articaine 4%, Septodont, France) for hemostasis. All the patients were anesthetized by the infiltration technique for the buccal mucoperiosteum and the infiltration technique for the palatal mucoperiosteum.

The proportions of autogenous bone to xenograft were not standardized across the groups, and the ratios were intentionally altered based on the unique biological and physical properties of the individual meshes. For the prebent titanium mesh group (control), the ratio of autogenous bone to xenograft was 60:40. This ratio is a well-established and validated approach in the literature for use with titanium meshes [15].

In the customized PEEK mesh group (study), a 70:30 combination of autogenous bone and xenograft material was used. This change was made to account for the decreased porosity and permeability of the PEEK material when compared to the titanium mesh material. It was thought that a higher percentage of autogenous osteogenic cells and factors would be necessary to encourage vascularization and graft incorporation through the less-permeable PEEK material. Although the difference in graft composition was deemed clinically relevant and necessary to optimize biological performance for each mesh type, it represents a methodological difference that precluded a direct and isolated assessment of the mesh material. Therefore, the present study should be interpreted as a comparison of two different treatment protocols, not the material itself as a separate entity.

**Fig. 2:**
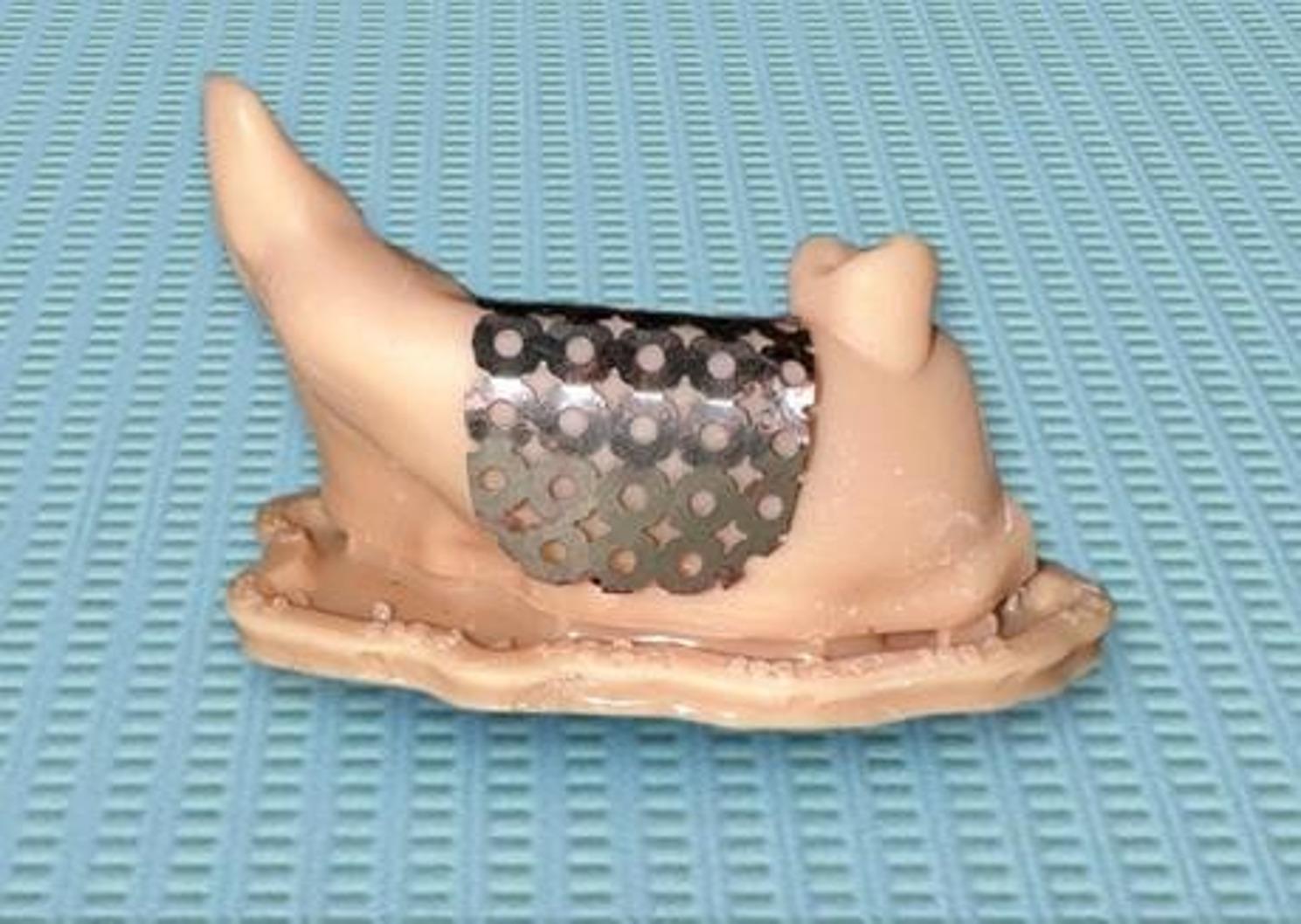
A stereolithographic (STL) model

Group 1 (control group): a stereolithographic (STL) model is designed from the reformatted DICOM files obtained from the CBCT, onto which the deficient site would be virtually augmented and 3-D printed. The Ti mesh would then be modified and adapted over the model before the surgery, as shown in Fig. 2.

### Stereolithographic (STL) model fabrication

**Fig. 3:**
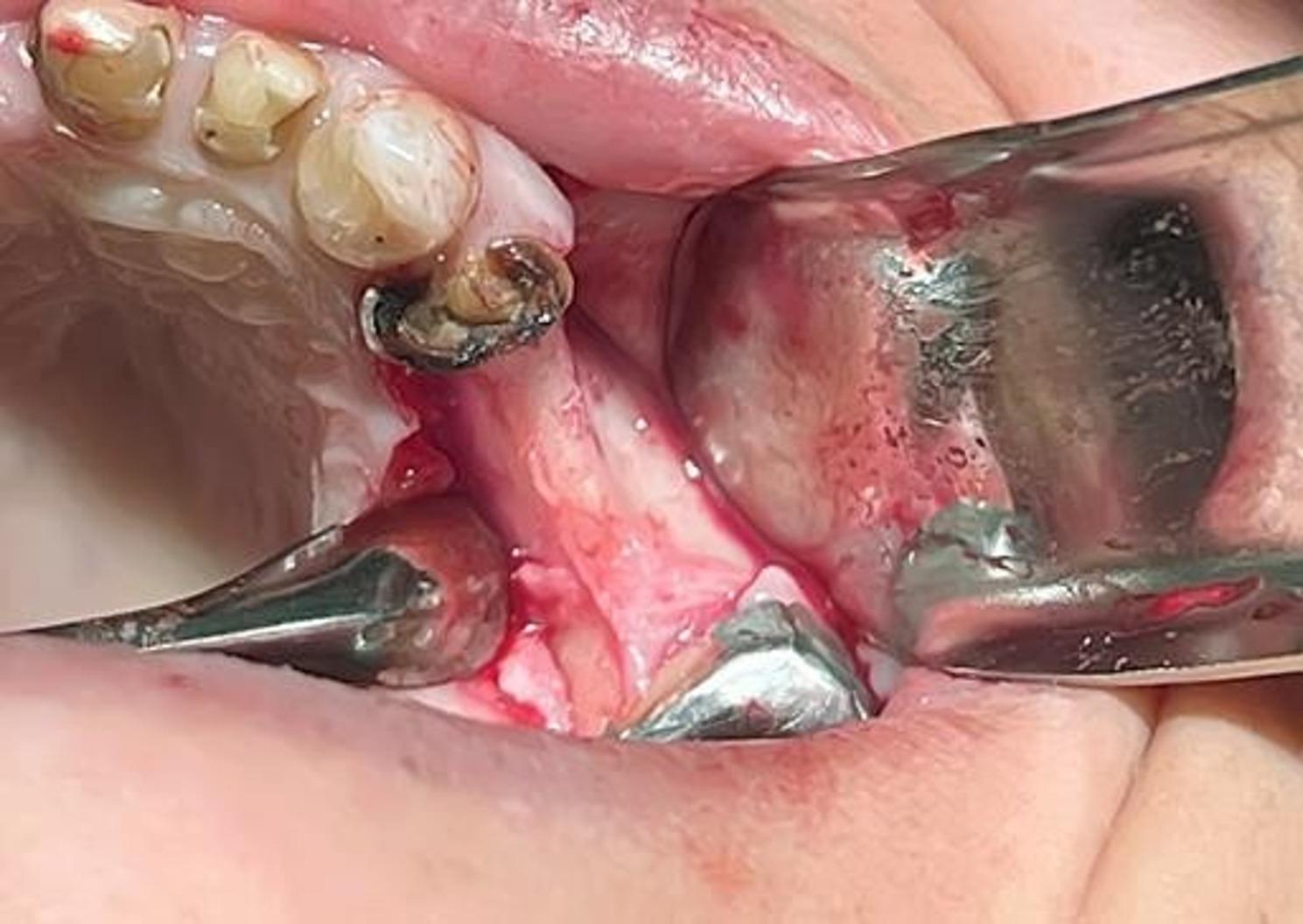
Full mucoperiosteal flap elevation

**Fig. 4:**
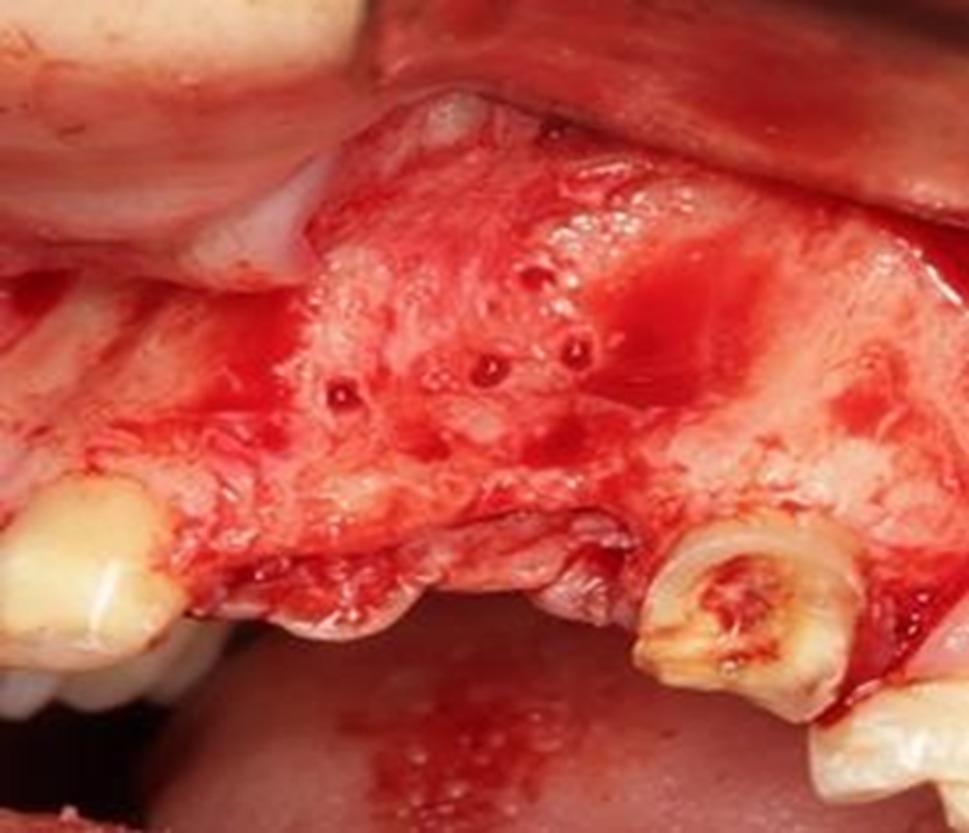
Decortication of the recipient site

**Fig. 5:**
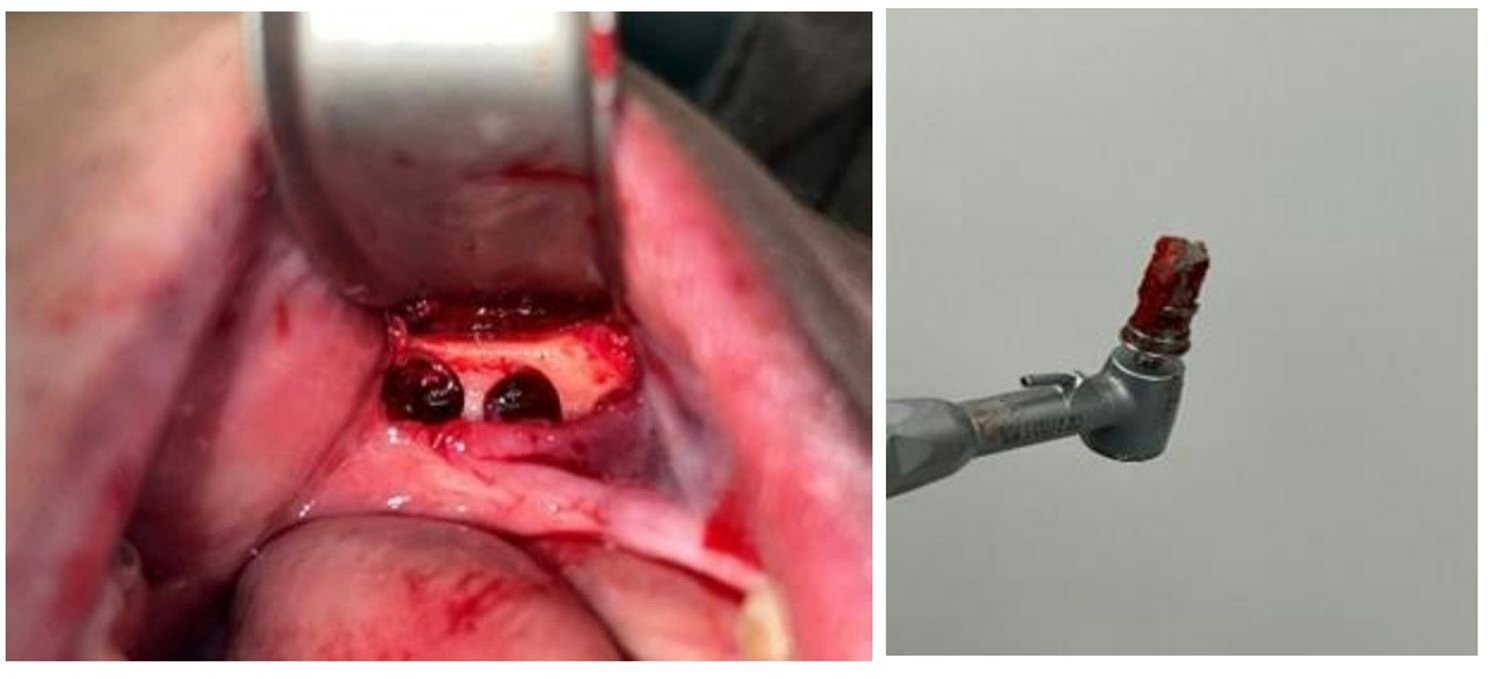
a and b Full mucoperiosteal flap of the donor site and harvesting bone with ACM BUR

**Fig. 6:**
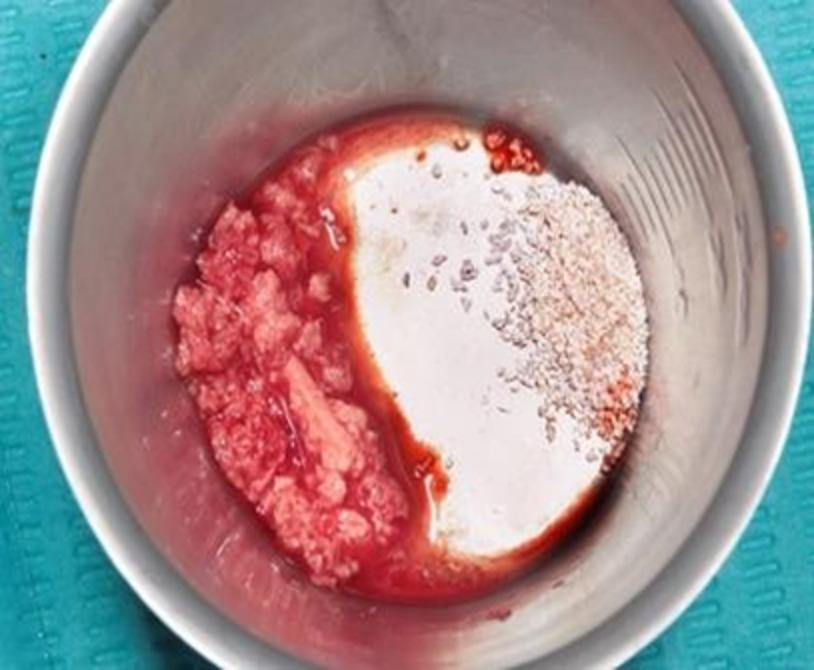
The bone graft ratio 60% autogenous and 40% xenograft

**Fig. 7:**
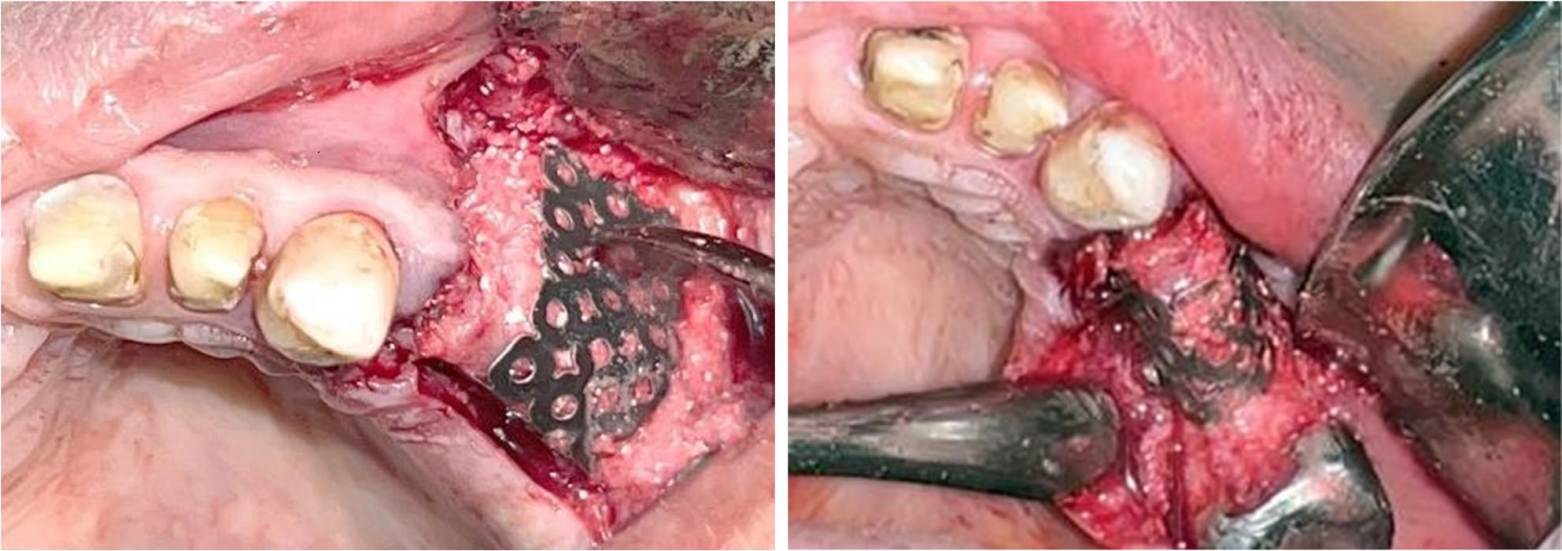
Ti mesh adaptation and fixation

**Fig. 8:**
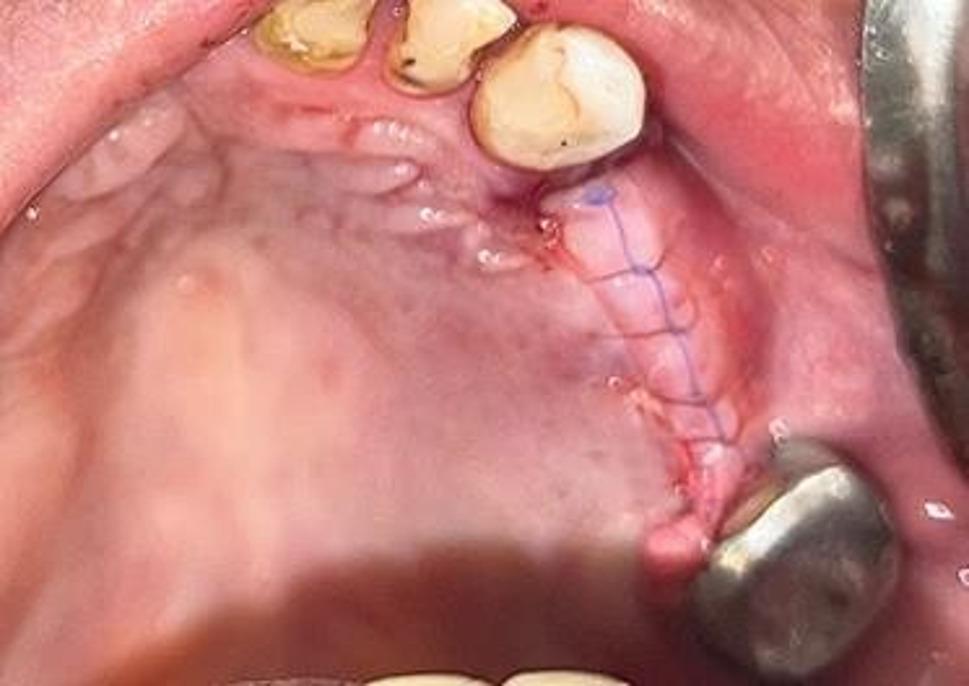
Flap suturing

1. A specific program (Mimics 19, Materialize NV, Belgium) was used to import DICOM files. In order to create a space for the particulate graft intraoperatively, the design process began with a virtual 3D (horizontal & vertical) incremental increase for the deficient ridge until acquiring the required dimensions. The virtually grafted 3D stereolithographic model was then created using 3D printing technology (Envisiontec GMBH, Gladbeck, Germany) as a guide for prebinding a readymade titanium mesh [5].
2. The alveolar bone was then exposed by raising a full-thickness mucoperiosteal flap, which comprised a para-crestal incision with one or two vertical releasing incisions, depending on the location and severity of the lesion. Next was a full-thickness reflection of the palatal and labial mucoperiosteal flaps [5]. Reflection was extended to expose the whole length of the facial cortical plate of the alveolar ridge, as shown in Fig. 3.

- To facilitate graft consolidation, bleeding sites (decortication) were performed using a tiny, rounded surgical bur to reveal the underlying marrow, as shown in Fig. 4.
- The donor site, which is the mandibular ramus, is then elevated after a full-thickness mucoperiosteal flap has been revealed. An ACM bur auto chip maker was used to extract particulate autogenous chips, as shown in Fig. 5a, and b.
- Then the autogenous bone was mixed with xenograft (60:40 mixture of autogenous bone and xenogeneic bone) as shown in Fig. 6.
- After the pre-bent titanium mesh was secured to the palatal side intraoperatively, a graft (a 60:40 blend of autogenous and xenogeneic bone) was applied. Three or four tiny titanium screws were then used to modify and secure the mesh, as shown in Fig. 7.
- Using a sharp 15 C surgical blade, a periosteal releasing incision was made on the flap’s underside for tension-free closure. Prolene sutures 4 − 0 were then used to close the flap, as shown in Fig. 8.

#### Group 2 (study group)

1.Depending on the position and size of the defect, one or two vertical releasing incisions were made in addition to a para-crestal incision, and full-thickness mucoperiosteal flaps were lifted to reveal the cortical bone. Next, the labial and palatal mucoperiosteal flaps were reflected in full thickness. The entire length of the alveolar ridge's facial cortical plate was revealed by extending the reflection.
To facilitate graft consolidation, bleeding sites (decortication) were created using a tiny, rounded surgical bur to reveal the underlying marrow.
After exposing a full-thickness mucoperiosteal flap for the donor location (the mandibular ramus), it is raised.

Particulate autogenous chips were harvested using an auto chip maker ACM bur.

**Fig. 9:**
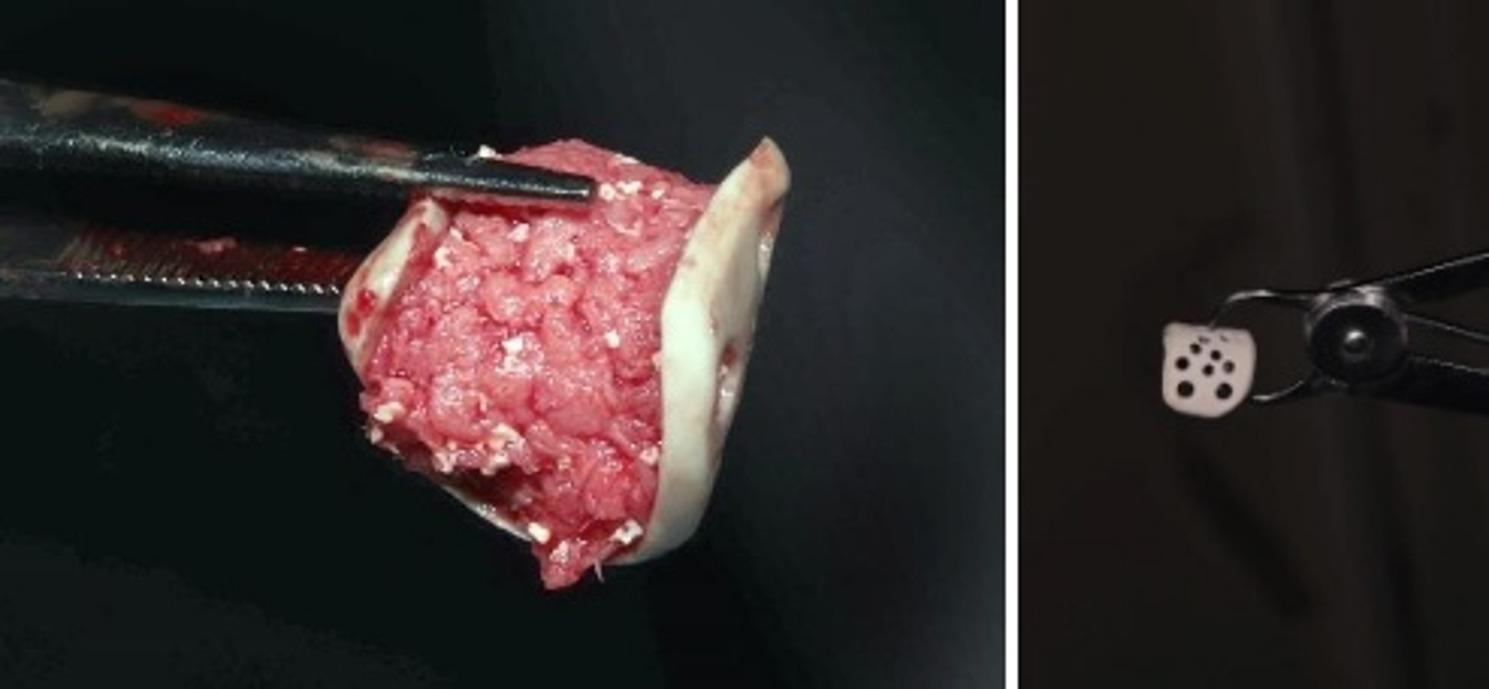
Loading of the PEEK mesh with the graft mixture

The PEEK mesh was loaded with the graft mixture, which is 70% autogenous bone and 30% xenograft, as shown in Fig. 9.

**Fig. 10:**
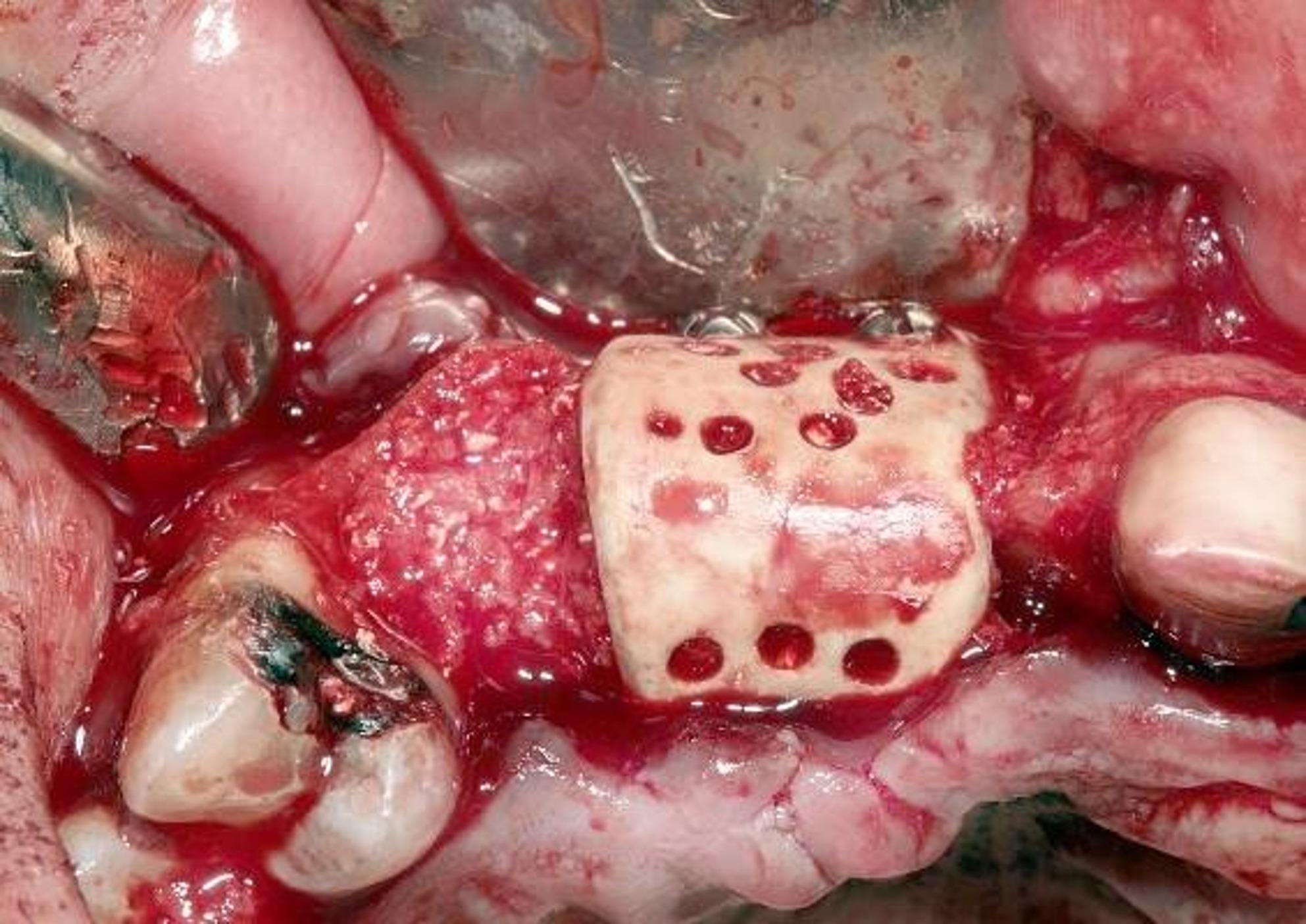
Fixation of the PEEK mesh with microscrews

At least three or four screws are used to secure the specially machined PEEK mesh in place, as shown in Fig. 10.

**Fig. 11:**
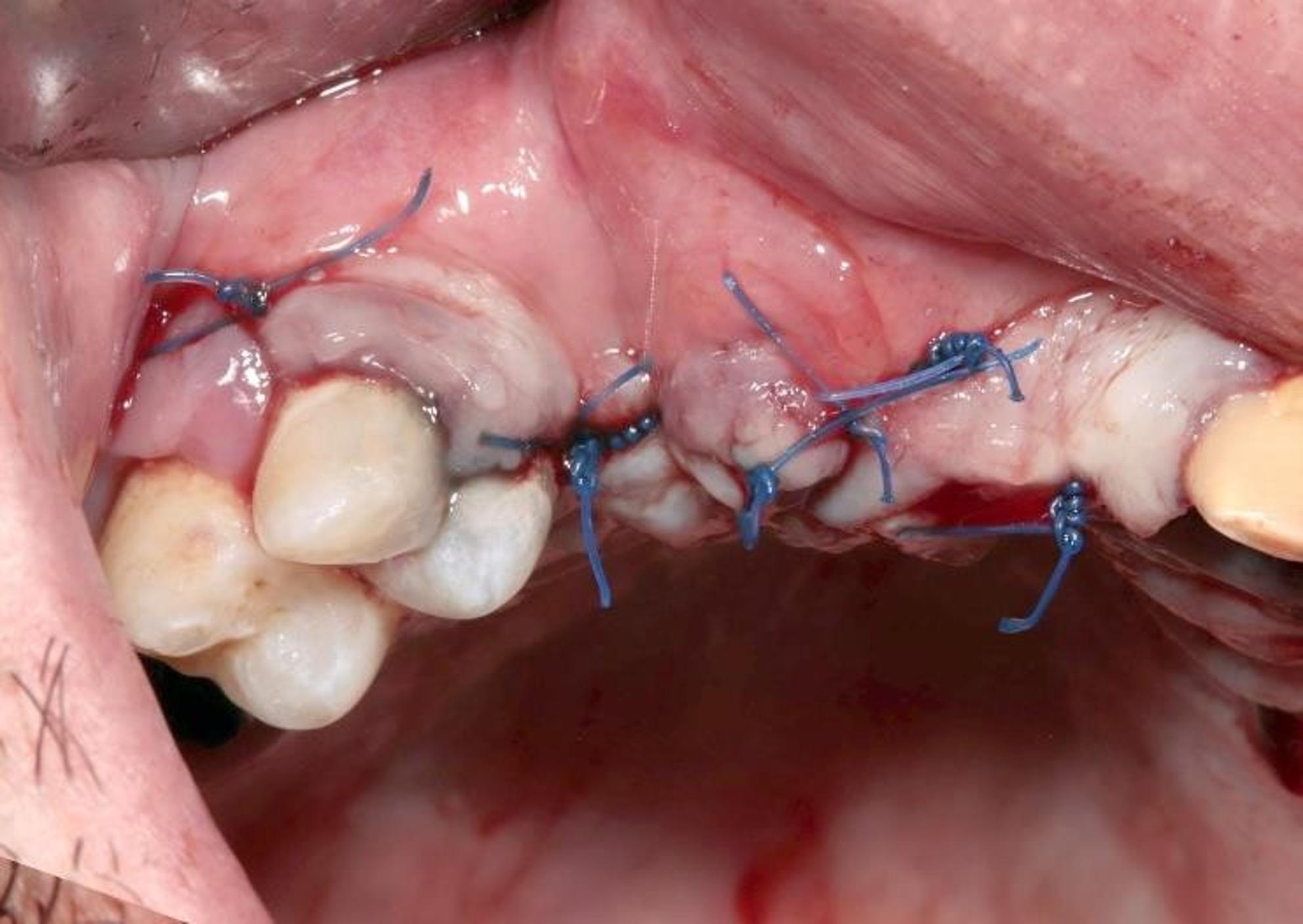
Flap suturing and closure

A sharp 15 C surgical blade was used to make a periosteal release incision on the flap's underside for tension-free closure. Proline 4-0 single interrupted sutures were then used to seal the flap, as shown in Fig. 11.

### PEEK mesh fabrication

**Fig. 12:**
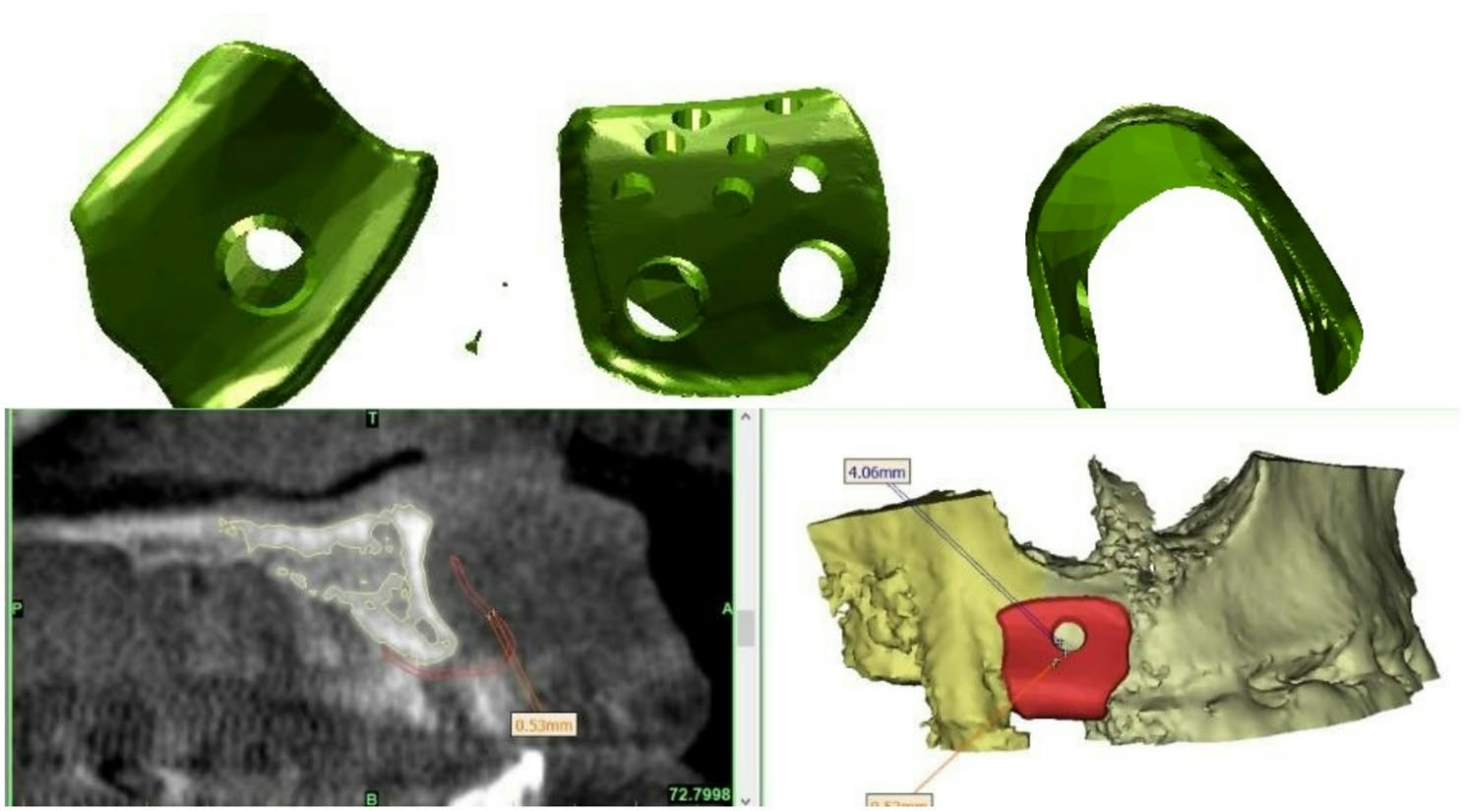
The patient customized PEEK mesh design ready for milling

As seen in Fig. 12, a 0.5 mm thick perforated meshwork was created to cover the buccal, crestal, and palatal sides of the alveolar bone. This allowed for the intended particle graft site to be placed between the fitting portion of the mesh and the resorbed native bone. Five five-axis milling machines were used to fabricate the final shape of the tailored device for this patient group, utilizing medically grade PEEK blocks (Juvora).

### Post-operative management

1. All patients were instructed to maintain optimal oral hygiene,
2. Avoid chewing solid textured food, and apply icepacks during the first day and then warm packs for the following two days
3. Use 0.12% chlorohexidine gluconate solution and betadine mouthwash as a mouth rinse daily for 2 weeks to aid in the maintenance of oral hygiene.
4. Patients were instructed to continue the antibiotic (Amoxicillin trihydrate 875mg + Clavulanate potassium 125mg) course for 5 days after surgery.
5. Ibuprofen 400mg was prescribed as an analgesic 3 times a day.
6. After 10 days, the sutures were removed.

### Postoperative follow-up

After 6 months, clinical evaluation of the operated site was carried out. A CBCT scan was done for all candidates to assess the amount of 3D bone gain, then a subperiosteal flap was reflected, the mesh was removed. After 6 months, CBCT evaluation was performed to assess the bone gain in three dimensions. Then, the mesh was removed, and implant placement was performed in the augmented sites.

### The Prosthetic phase

After 4 months, a crestal incision was made to expose the implant’s platform, and a healing abutment was attached to each implant, and then the soft tissue was sutured around it.

After two weeks, an open tray impression technique was used to fabricate cement-retained porcelain-fused-to-metal (PFM) restorations.

**Fig. 13:**
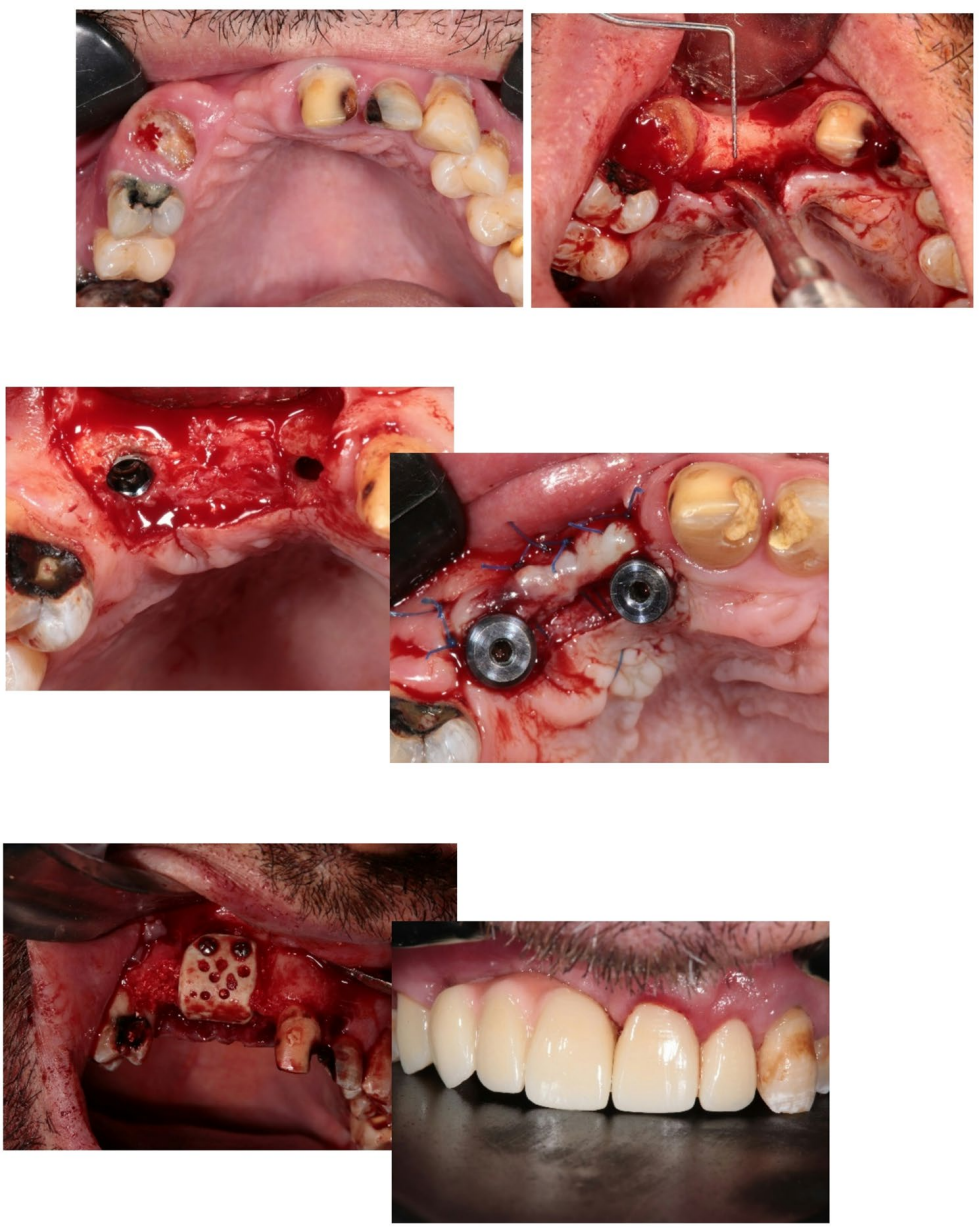
**A**–**F** Sequential clinical presentation of surgical and prosthetic management. **A** Preoperative condition. **B** Flap elevation and defect exposure. **C** Osteotomy preparation. **D** Implant placement. **E** Ridge augmentation using guided bone regeneration. **F** Final prosthetic rehabilitation

A complete case history is illustrated in Figures (13 A–F), showing the stepwise approach towards surgery and prosthesis for the defect, starting from initial presentation to the final stage of rehabilitation.

### Statistical analysis

The categorical data was expressed in the form of frequency and percentage values, and the analysis was done using Fisher’s exact test. The numerical data was expressed in the form of mean and standard deviation values, and the normality of the data was checked using the Shapiro-Wilk test.

Randomization was performed at the patient level, while measurements of the outcomes were taken at the site level, where some individuals had multiple sites. Hence, the observations at the site level were not independent.

As some patients had more than one site, the data was not independent for the augmented sites. The analysis was done using the linear mixed-effects model, where the treatment (titanium or PEEK) was included as the fixed effect, and the patient was included as the random effect.

More specifically, a random intercept was added per each patient in order to allow for intra-patient clustering of sites. No other site-level covariates were included in the model. The assumption of model normality and equal variances were tested via graphical methods and deemed to be satisfied.

The level of significance was fixed at 0.05, and the analysis was done using the R statistical software package, version 4.4.1.

Descriptive statistics are provided at the site level, while for the inferential comparisons were performed using mixed-effect models accounting for clustering within patients. All analyses accounted for the hierarchical structure of the data (sites nested within patients).

## Results

In the present study, all the outcome comparisons between groups were performed by linear mixed effects models to adjust for clustering within patients who had multiple sites.

### Demographic data

The study was conducted on 14 patients with 28 augmented sites; the patients were equally and randomly allocated to both groups (14 sites per group). Each patient contributed one or two sites, and no patients or sites were lost to follow-up, and all randomized participants were included in the final analysis. No protocol deviations occurred during the study.

In the control group, there were 2 males and 5 females with a mean age of (38.89 ± 6.26) years. In the study group, there were 4 males and 3 females with a mean age of (34.33 ± 5.92). There was no significant difference between the two groups regarding gender (*p* = 0.592) and age (*p* = 0.187), as shown in Table 1.

**Table 1:** Summary statistics for demographic data

Parameter		Control	Study	*p*-value
Gender [n (%)]	Male	2 (28.57%)	4 (57.14%)	**0.592ns**
	Female	5 (71.43%)	3 (42.86%)	
Age (Mean ± SD) (years)		38.89 ± 6.26	34.33 ± 5.92	**0.187ns**

*ns* not significant

### Clinical results

Postoperative outcomes for patients undergoing maxillary bone augmentation with titanium and PEEK meshes were largely positive, with minimal complications observed.

All patients experienced postoperative edema, which resolved entirely within one week of the procedure, indicating a typical recovery profile. Importantly, there were no infections reported at the donor site across all cases, underscoring the overall procedural safety in both groups.

Mesh exposure was noted in 2 cases (14.3%) in the titanium group and 1 case (7.1%) in the PEEK group, with no statistically significant difference between groups (Fisher’s exact test, *p* > 0.05).

Severe bleeding occurred in 1 case in the titanium group; however, there were no cases in the PEEK group (*p* > 0.05). No neurosensory problems were noted in either group. Overall, the clinical outcomes indicate both materials are well-tolerated, with low complication rates observed in both groups as presented in Table 2.

**Table 2:** Summary of postoperative complications

Complication	Titanium (*n* = 7)	PEEK (*n* = 7)
Postoperative edema	7 (100%)	7 (100%)
Infection (donor site)	0 (0%)	0 (0%)
Mesh exposure	2 (14.3%)	1 (7.1%)
Timing of exposure	4–5 months	3 months
Severe bleeding	1 (14.3%)	0 (0%)
Neurosensory disturbance	0 (0%)	0 (0%)
Implant placement success	7 (100%)	7 (100%)

### Vertical bone gain

**Fig. 14:**
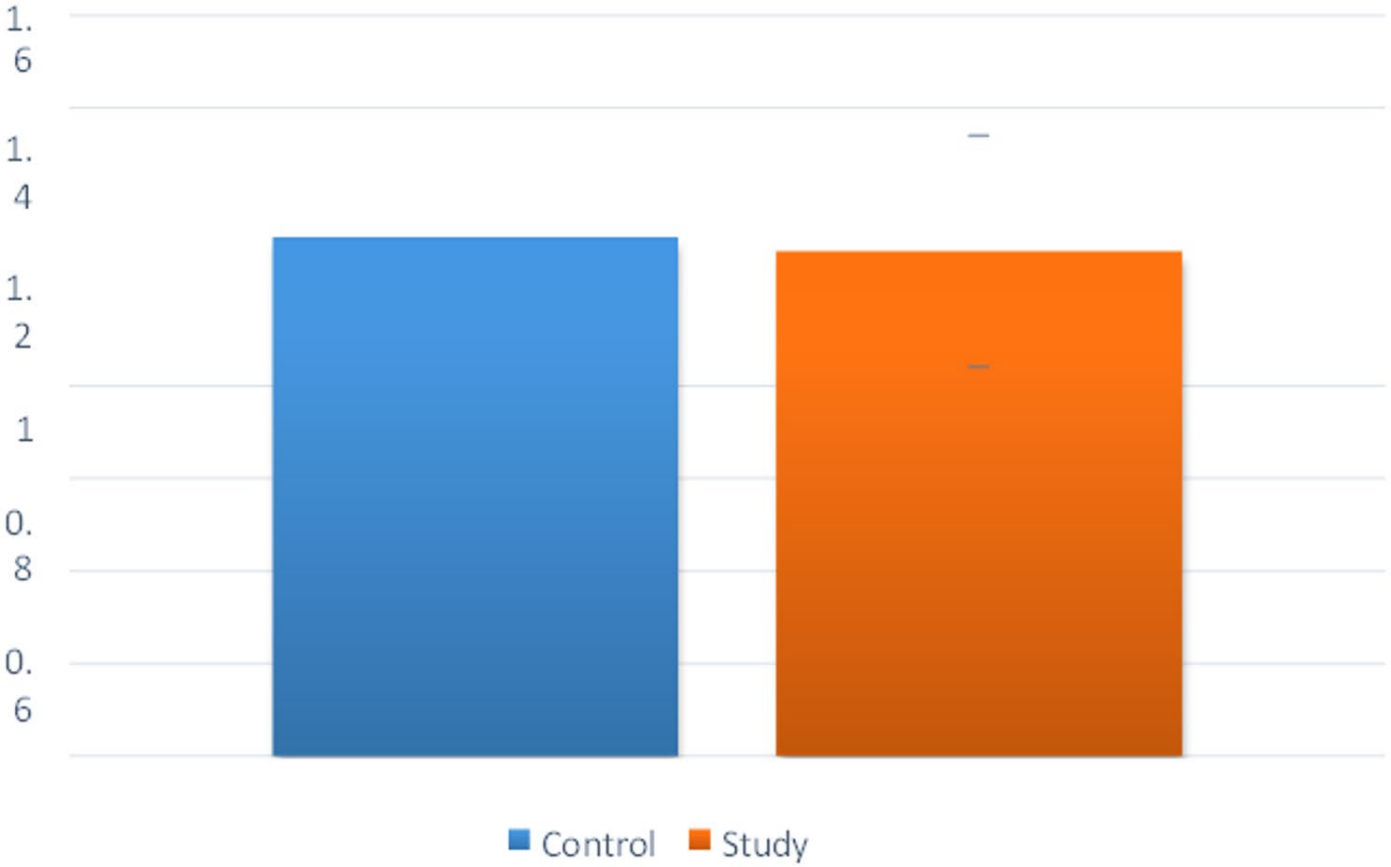
Bar chart showing mean and standard deviation values of vertical bone gain (mm)

Vertical bone gain measured in the control group (1.12 ± 0.10) (mm) was higher than that of the study group (1.09 ± 0.25) (mm). However, the difference was not statistically significant (*p* = 0.738), as shown in Fig. 14.

### Horizontal bone gain

**Fig. 15:**
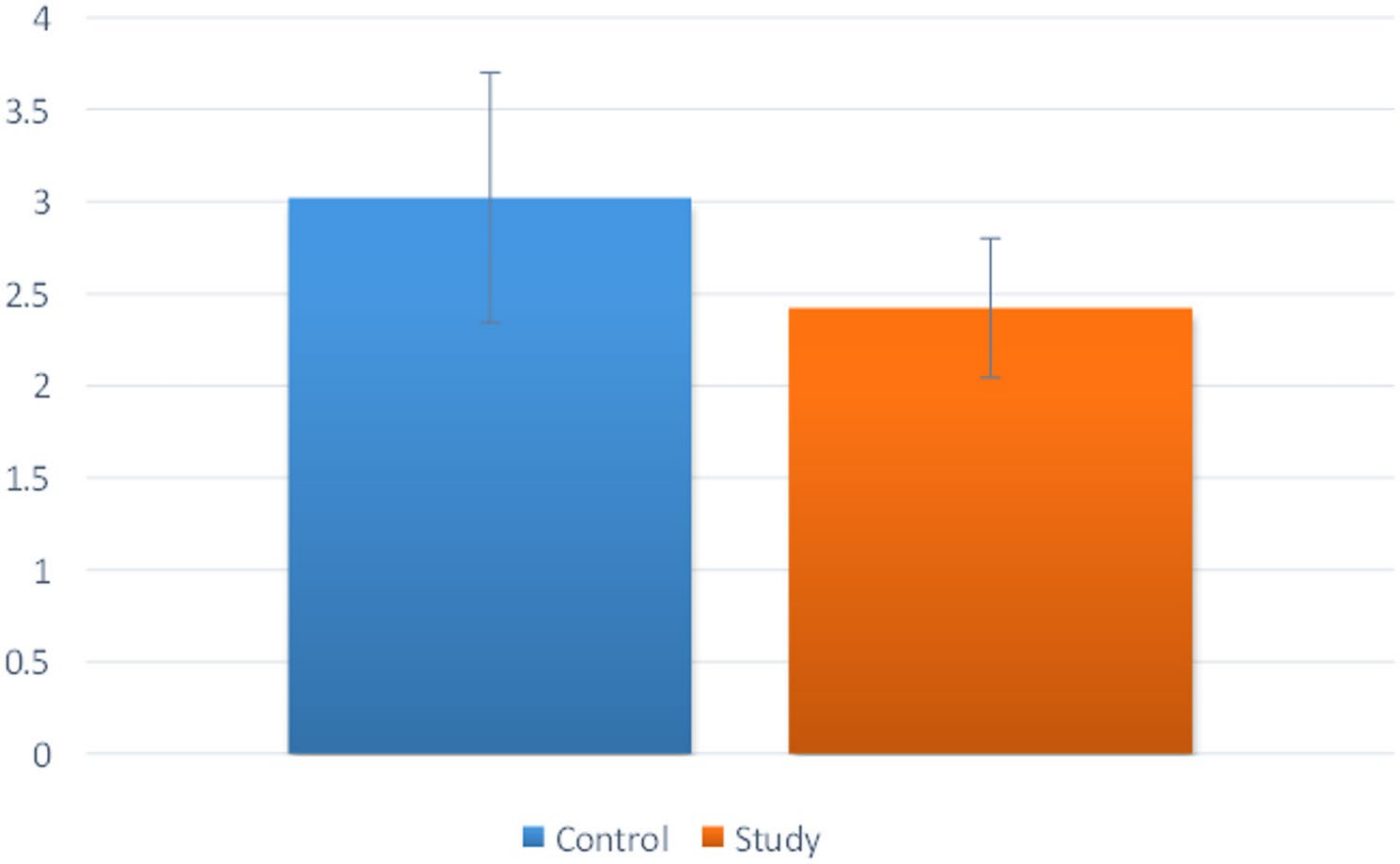
Bar chart showing mean and standard deviation values of horizontal bone gain (mm)

Horizontal bone gain measured in the control group (3.02 ± 0.68) (mm) was higher than that of the study group (2.42 ± 0.38) (mm). However, the difference was not statistically significant (*p* = 0.065), as shown in Fig. 15.

### Gained bone volume

**Fig. 16:**
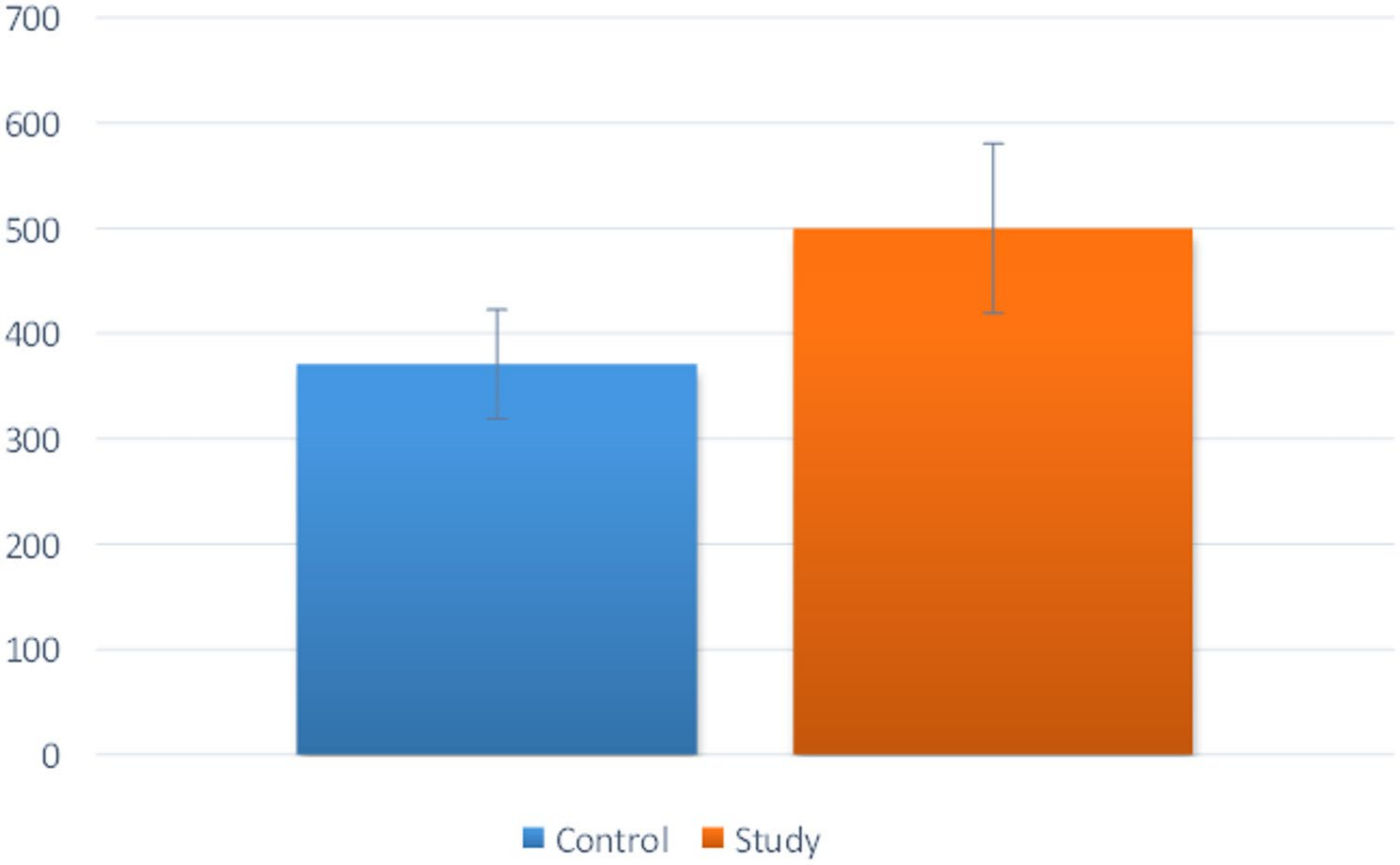
Bar chart showing mean and standard deviation values of gained bone volume (mm^3^)

The gained bone volume measured in the study group (499.47 ± 80.46) (mm3) was significantly higher than that of the control group (370.82 ± 51.69) (mm3) (*p* = 0.004), as shown in Fig. 16.

### Graft loss volume

**Fig. 17:**
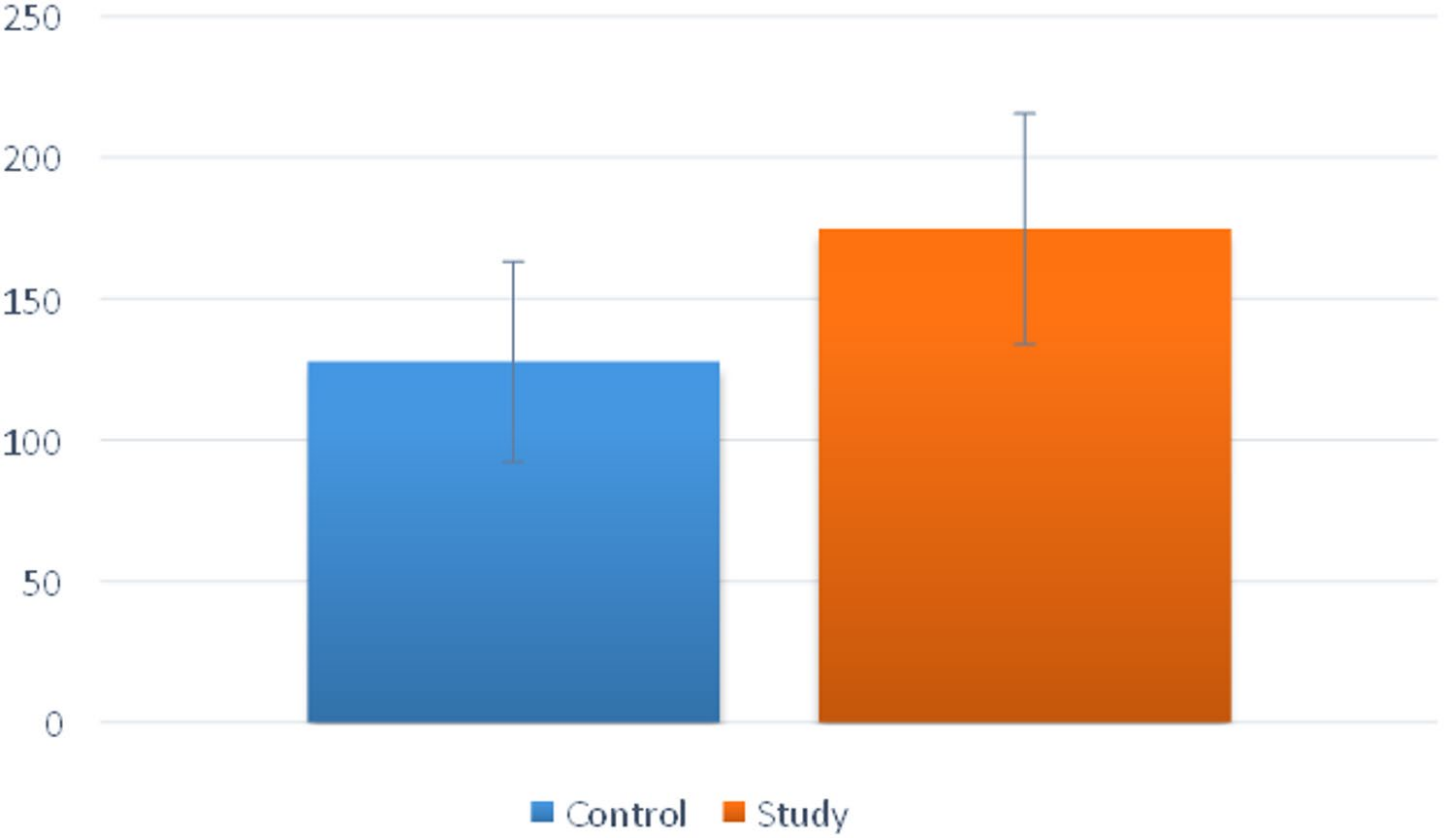
Bar chart showing mean and standard deviation values of graft loss volume (mm^3^)

The graft loss volume measured in the study group (174.83 ± 40.78) (mm3) was significantly higher than that of the control group (127.73 ± 35.55) (mm3) (*p* = 0.040), as shown in Fig. 17.

It is interesting to note, however, that the percentage volume of graft loss between the two groups was almost similar (34% for the titanium group and 35% for the PEEK group).

## Discussion

The integration of dental implants in oral rehabilitation to achieve optimal functionality, aesthetics, and longevity has become a standard practice. Successful implant placement relies on several factors, including the presence of adequate three-dimensional bone volume, which is mandatory for appropriate implant bone support, and appropriate implant angulation to ensure long-term implant success.

However, alveolar ridge defects arising from conditions like periodontal disease, trauma, prolonged edentulism, and congenital anomalies often render implant placement impossible.

To address this challenge, the alveolar bone should initially be reconstructed to allow for subsequent successful implant placement.

Several techniques have been reported in the literature for alveolar bone regeneration, such as block graft and guided bone regeneration (GBR) using either resorbable or non-resorbable membranes. Every protocol has advantages and disadvantages, and it should be customized based on the demands of each patient, the operator’s expertise, and personal preferences.

Guided bone regeneration using non-resorbable membranes offers several advantages, including the ability to augment large bone defects on the one hand; block grafts offer only limited volume, which can be augmented owing to the limited availability of the donor sites. Moreover, it tends to offer the ability to create and maintain a stable and well-defined volume, unlike non-resorbable membranes, where it is difficult to maintain a well-defined volume [2].

Titanium mesh has been applied with great success in guided bone regeneration procedures owing to its favorable characteristics such as malleability, flexibility, strength, rigidity, and biocompatibility. Despite its merits, titanium mesh requires additional intraoperative adjustment to customize it, increased risk of soft tissue dehiscence owing to the created sharp edges from the trimming, and inability to perform the required smoothness chair-side. Hence, with the aid of CAD/CAM technology, a virtually augmented model could be created for adjusting and pre-bending the titanium mesh accordingly [3].

The creation of a virtually augmented three-dimensional model ensures that the augmentation procedure will create the precise three-dimensional contour for successful implant placement without over or under-grafting. Furthermore, this helps to reduce patient morbidity since only the required amount of graft would be harvested. Injury to adjacent structures, such as neighboring teeth or the infraorbital nerve, during adaptation and fixation of the titanium mesh can easily be avoided. Finally, preoperative adjustment and prebinding of the titanium mesh allow the surgeon to ensure superior smoothness of the mesh, thus reducing the probability of soft tissue dehiscence postoperatively [16].

Recent advancements in digital workflow have further improved the effectiveness of customized titanium mesh in guided bone regeneration procedures. De Santis et al. (2021) [12] have shown the effectiveness of digitally designed patient-specific titanium mesh in treating vertical, horizontal, and combined defects. Recently, De Santis et al. (2024) [17] have proposed an innovative method for designing customized titanium mesh, with particular emphasis on optimization of mesh geometry and soft tissue management. This shows the increased emphasis on digital workflow for improved predictability in guided bone regeneration procedures.

Several combinations and proportions of grafting materials have been reported in the literature. The graft material used in our study was composed of a mixture of autogenous bone and xenograft, with ratios differing between groups (60:40) in the titanium group and (70:30) in the PEEK group [15]. The graft mixture offers osteogenic potential owing to living osteoblasts from the harvested autogenous bone, as well as osteoinductive properties, while reducing patient morbidity, as well as delaying graft resorption due to the presence of xenograft, allowing for complete regeneration of the newly created volume [16].

It is noteworthy that this study should be interpreted as a comparison between two types of treatment protocols, not simply as a comparison between mesh materials since the difference between the groups concerning the graft composition is a major consideration.

This study primarily focused on horizontal ridge deficiencies, with only limited vertical augmentation achieved, as indicated by the relatively small vertical gain values.

Recent advancements in biomaterials have shown that PEEK material is a potential alternative approach in maxillofacial reconstruction, due to its biocompatibility, reduced soft tissue irritation, and suitable mechanical properties. The advent of CAD/CAM technology has been used in a few studies attempting to use PEEK as a barrier membrane in guided bone regeneration [6].

The use of customized PEEK meshes has been associated with potential clinical advantages, such as improved adaptation and more favorable soft tissue reaction, which may contribute to decreasing the rates of membrane exposure [18].

However, the design and application of PEEK mesh require precise preoperative planning, high precision in design and fabrication, since intraoperative modifications are limited. The relatively increased thickness of the material mandates that the surgeon must take it into account in the design phase, and may require more extensive soft tissue release to ensure tension-free closure over the grafted site [18].

Mounir et al. [5] were one of the first to report on the use of PEEK mesh in guided bone regeneration. He reported that a thickness of 2 mm was determined for the mesh to prevent fracture. In the current study, the authors set the mesh thickness to 0.7 mm to reduce the risk of soft-tissue dehiscence at the grafted site. No case of membrane fracture was noted in our study, showing that a 1 mm PEEK membrane thickness is adequate for fixation [5, 7].

Di spirito et al. [19], have reported that the incidence of titanium mesh exposure varied from 20% to 66%. In the current study, titanium mesh exposure was noticed in two augmented sites. The first exposure was noted at 4 months postoperative, while the second was noted at 5 months postoperative. Both cases were managed conservatively, with strict oral hygiene instructions via daily saline irrigation and chlorohexidine mouthwash. The delayed exposure of the mesh allowed the graft to consolidate and reduced the risk of total graft loss.

This finding is consistent with previous clinical reports and narrative reviews indicating that mesh exposure does not necessarily compromise bone regeneration if the biomaterial remains stable and infection is prevented [19].

Regarding the PEEK group, PEEK mesh exposure was recorded in one patient that occurred on the third month postoperatively; however, this complication did not affect the outcome as it was successfully managed conservatively with daily Chlorohexidine and saline irrigation and strict oral hygiene measures. This favorable response can be attributed to the smoothness of the external surface of the milled PEEK mesh [5].

Thus, the use of mixed effects modeling provides a more accurate basis for making statistical inferences due to the proper accounting for within-patient correlation.

In the current study, the titanium mesh group showed an average bone gain of 3.02 ± 0.68 mm and an average vertical gain of 1.12 ± 0.10 mm. The average bone gain in our study was less than that reported in the literature, where a 5 mm average horizontal gain and up to 7 mm average vertical gain were reported [3, 4].

Concerning the PEEK mesh group, the average bone gained horizontally was 2.42 ± 0.38 mm, and the average vertical bone gained was 1.09 ± 0.25 mm. These results demonstrated that both membranes were capable of achieving bone regeneration within the conditions of this study.

In the current study, we present a comparative assessment of the gained bone volume with both augmentation techniques and also the volume of graft resorption at 6 months postoperatively. The gained bone volume measured in the PEEK group was 499.47 ± 80.46 mm³, which was statistically significantly higher than that of the titanium group, 370.82 ± 51.69 mm³.

Although the PEEK group had a significantly greater absolute bone gain, it also had a higher amount of graft loss. However, when graft resorption was expressed as a percentage of the initial augmented volume at baseline, comparable values were observed between both groups (around 34–35%). Hence, it can be presumed that the greater graft loss in the PEEK group is likely proportional to the initially larger augmented volume rather than indicative of inferior graft stability.

However, these results should be interpreted cautiously because it is not possible to attribute these differences entirely to the material used in the mesh, due to differences in composition among the groups.

One of the most important methodological considerations in the present study is the difference in graft composition used in the two groups. It is well established that the proportion of autogenous bone plays a critical role in bone regeneration due to its osteogenic, osteoinductive, and osteoconductive properties. Histological and histomorphometric studies have shown that the process of bone formation is not only related to the barrier device but also to the composition of the graft used, as well as the vascularization dynamics [3, 11, 20].

The higher proportion of autogenous bone used in the PEEK group (70:30) may have enhanced initial bone formation, but may also have contributed to increased remodeling and resorption, which could explain the greater graft loss observed. Hence, the differences in volumetric outcomes could not be attributed to the mesh material alone, but rather to the combined effect of graft composition and barrier characteristics.

In addition, although all defects met the predetermined inclusion criteria, detailed baseline defect dimensions were not analyzed for each group separately. Therefore, potential differences in the initial defect size or planned augmentation volume of the defects cannot be excluded, which may have influenced the volumetric outcomes.

Similarly, the graft loss volume measured in the PEEK group was 174.83 ± 40.78 mm³, which was statistically significantly higher than that of the titanium group, 127.73 ± 35.55 mm³. This finding may be partly explained by the lower porosity of the PEEK mesh that could limit vascular penetration. Additionally, the higher autogenous bone ratio used in this group (70:30) may have contributed to increased remodeling and resorption. Differences in material properties, such as mesh porosity, may be a contributing factor. Nevertheless, these interpretations should be considered as hypothetical, as the current study does not allow for direct evaluation of the exact underlying mechanism.

On the other hand, Mounir et al. found that there is no significant difference between the groups concerning the graft loss. This could be explained by the lack of standardization in the PEEK mesh design regarding the amount and dimensions of the pores in the mesh [6].

The current study findings align with previous research, demonstrating that both materials are effective in promoting bone regeneration. While titanium mesh offers affordability and availability, its rigidity and tendency to form sharp edges pose clinical challenges [6].

Therefore, volumetric outcomes should be interpreted cautiously, as graft composition may have independently influenced resorption dynamics.

Another parameter that can be useful in improving the assessment of digital-assisted bone regeneration techniques is the regeneration rate (RR), which can be calculated by dividing the volume of the newly formed bone by the planned volume of the bone augmentation. The usefulness of this parameter in assessing the accuracy of bone augmentation procedures has been emphasized. However, in the present study, the volume of the bone augmentation has not been quantitatively measured in the digital workflow. Therefore, it has not been possible to calculate the RR. It should be considered in future studies for more precise volumetric evaluation.

Other factors, such as pseudoperiosteum features and bone resorption patterns, may offer further information on the biological quality of the newly formed bone tissue. Previous studies have highlighted the relevance of pseudoperiosteum classification in assessing the quality of tissue integration in soft tissue [20], in addition to the influence of bone resorption on the stability of the bone tissue [21].

Moreover, clinical studies involving customized meshes have emphasized the significance of biological assessment for the evaluation of regenerative outcomes [22]. However, these variables were not considered in the present study, as they need to be specifically assessed by clinical or histological protocols and need further time points for evaluation. Such variables should be considered in future studies for a better evaluation of regenerative outcomes.

The customized nature of PEEK meshes may offer potential advantages; however, these aspects were not directly examined in this clinical trial. Therefore, further investigations are needed to assess the clinical effectiveness of PEEK meshes and optimize the criteria for fabrication and application as a barrier membrane.

Additionally, the development of a standardized finishing protocol for PEEK is needed to ensure consistent surface quality and biocompatibility. Finally, the choice between titanium and PEEK mesh should be guided by patient-specific factors, anatomical challenges, and the surgeon’s preference.

One of the major considerations when interpreting the results of this study is the graft composition, which differed between the two groups. The titanium mesh group was supplied with a graft composition of 60:40, while the PEEK mesh group was supplied with a graft composition of 70:30. The rationale behind this was clinical, driven by the need to provide the optimal biological environment for each material, particularly in the case of the less porous material, the PEEK. The lack of standardization, however, introduces a major confounding variable.

Consequently, the differences in the volume of bone obtained and the resorption rate cannot be entirely attributed to the mesh material, but it appears to be the combined effect of both the mesh material and the graft material. It is important to note that this study was not intended to be a direct comparison of the materials, but it can be viewed as a comparison of the efficacy of two different treatment modalities, i.e., Mesh A + Graft A, and Mesh B + Graft B.

The conclusions about postoperative complications drawn from this study require careful consideration. With a modest number of patients involved and the infrequent occurrence of complications in this patient group, there is insufficient statistical power to determine differences between the groups. The study did not have sufficient power to analyze outcomes related to complications.

### Limitations

Although this randomized controlled trial offers relevant comparative information regarding the use of pre-bent titanium and custom-made PEEK meshes for three-dimensional maxillary ridge augmentation, certain limitations have to be considered. First, the relatively limited sample size. Although the sample size was calculated based on the primary outcome, which is the horizontal bone gain, the study might be underpowered to detect the difference in secondary outcomes such as postoperative complications and volumetric changes. Therefore, the results of the complications analysis should be interpreted with caution, and the absence of statistically significant differences does not mean equivalence of the two treatment protocols. Larger randomized controlled trials are needed to confirm these results. Third, since follow-up assessments were conducted for only six months following augmentation procedures, information regarding bone gain and resorption could be obtained for the early phase following augmentation, but long-term outcomes regarding successful implant placement and crestal bone stability were not acquired.

Fourth, the single-center design does introduce site-specific biases regarding surgical technique, patient population, and postoperative care protocols. Fifth, the absence of standardization in the composition of the graft in the two groups is a limitation. Although the percentage of autogenous bone in the PEEK group was justified to compensate for the lower porosity of the PEEK mesh, it is apparent that the difference in the composition of the graft in the two groups created a confounding variable. Therefore, the observed results reflect the combined effect of the composition of the mesh and the composition of the graft. The results obtained in the study cannot be solely attributed to the mesh material. A further limitation of the present study is the absence of histological evaluation, including the assessment of pseudoperiosteum formation and early bone resorption patterns, which could provide further insight into the biological quality and maturation of the regenerated bone.

Furthermore, although advanced, CBCT volumetric analysis naturally has inherent limitations in both segmentation accuracy and threshold sensitivity. Additionally, the lack of surgeon blinding was due to the distinct physical properties of the two meshes, thus introducing performance bias.

Another limitation is the lack of detailed baseline defect characteristization at the site level. Although, all included defects in this study met the predefined inclusion criteria in terms of horizontal ridge width, but there was no systematic measurement done for the baseline horizontal ridge width, vertical deficiency, and defect volume between groups. This would limit the ability to assess baseline comparability after randomization and could potentially affect the interpretation of volumetric results, which are necessarily dependent upon the extent of the defect.

Finally, this study did not assess economic factors or logistical considerations, such as cost, fabrication time, and clinical availability, which are relevant to real-world adoption.

### Practical and clinical implications

Several clinical implications for practicing surgeons engaged in alveolar ridge reconstruction arise from this study. Both pre-bent titanium and patient-customized PEEK meshes were successful in both bone dimension increments. In practice, clinicians could consider particular specifics of cases when selecting between these materials. Thus, although PEEK meshes generated a greater bone volume, possibly due to precise graft design before surgery, they also had greater grafts of bone resorption, possibly due to low vascular permeability. Patient-customized titanium meshes are a proven and economical option with established safety. Their adaptation is very important during surgery to avoid soft tissue issues.

Accuracy may be enhanced and prosthetic-driven outcomes improved through the integration of CAD/CAM technology in planning both modalities. From a practical perspective, the use of customized PEEK meshes may be particularly favorable in cases of anatomical complexity or when there is a need to minimize mucosal irritation. However, surgeons must continue to provide considerable soft tissue release to accommodate the thicker design of PEEK mesh and remain cognizant that intraoperative adjustments in mesh configuration are not possible. Standardization of pore design in PEEK meshes, optimizing graft protocols, and long-term comparative studies to confirm these early findings in wider clinical practice are efforts that should be undertaken in the future.

Conclusions cannot be made in relation to the advantages of one mesh over the other on the basis of complication rates alone. Larger clinical trials will have to be conducted for this purpose.

In conclusion, both titanium and custom-made PEEK meshes were associated with successful horizontal and vertical ridge augmentation, enabling subsequent implant placement in all cases within the limitations of this study.

There were no statistically significant differences were observed between groups regarding the horizontal and vertical bone gain. While differences in volumetric outcomes between groups were observed, these results should be interpreted cautiously since they may not be entirely attributable to the mesh materials alone, given the difference in graft composition between groups.

Therefore, the present study should be interpreted as a comparison between two different treatment protocols, each combining a specific mesh material with a distinct graft composition, rather than a direct comparison of mesh materials alone. No definitive conclusions can be drawn regarding the superiority of either material.

## Data Availability

Research data supporting this publication is available from the corresponding author upon request.
